# Dosimetric feasibility of computed tomography-based image-guided brachytherapy in locally advanced cervical cancer: a Japanese prospective multi-institutional study

**DOI:** 10.1093/jrr/rraa138

**Published:** 2021-02-03

**Authors:** Yuki Otani, Tatsuya Ohno, Ken Ando, Kazutoshi Murata, Shingo Kato, Shin-ei Noda, Keiko Murofushi, Hiroki Ushijima, Daisaku Yoshida, Noriyuki Okonogi, Fumiaki Isohashi, Masaru Wakatsuki, Takashi Nakano

**Affiliations:** Department of Radiation Oncology, Osaka University Graduate School of Medicine, 2-2 (D10) Yamada-oka, Suita, Osaka 565-0871, Japan; Department of Radiology, Kaizuka city hospital, 3-10-20 Hori, Kaizuka, Osaka, 597-0015, Japan; Department of Radiation Oncology, Gunma University Graduate School of Medicine, 3-39-22 Showa-machi, Maebashi, Gunma 371-8511, Japan; Department of Radiation Oncology, Gunma University Graduate School of Medicine, 3-39-22 Showa-machi, Maebashi, Gunma 371-8511, Japan; Department of Radiation Oncology, Gunma Prefectural Cancer Center, 617-1 Takabayashinishi-machi, Ota, Gunma 373-8550, Japan; Department of Radiation Oncology, Gunma University Graduate School of Medicine, 3-39-22 Showa-machi, Maebashi, Gunma 371-8511, Japan; Department of Radiation Oncology, Saitama Medical Univercity International Medical Center, 1397-1 Yamane, Hidaka, Saitama 350-1298, Japan; Department of Radiation Oncology, Saitama Medical Univercity International Medical Center, 1397-1 Yamane, Hidaka, Saitama 350-1298, Japan; Department of Radiation Oncology, University of Tsukuba Hospital, 2-1-1 Amakubo, Tsukuba, Ibaraki 305-8576, Japan; Department of Radiation Oncology, Tokyo Metropolitan Cancer and Infectious diseases Center Komagome Hospital, Honkomagome 3-18-22 Bunkyo, Tokyo 113-8677, Japan; Department of Radiation Oncology, Saitama Cancer Center, 780 Komuro, Ina, Kita Adachi-gun, Saitama 362-0806, Japan; Department of Radiation Oncology, Saku Central Hospital Advanced Care Center, 3400-28 Nakagomi, Saku, Nagano 385-0051, Japan; Department of Radiation Oncology, Kanagawa Cancer Center, 2-3-2 Nakao, Asahi-ku, Yokohama 241-8515, Japan; QST Hospital, National Institutes for Quantum and Radiological Science and Technology, 4-9-1 Anagawa, Inage-ku, Chiba 263-8555, Japan; Department of Radiation Oncology, Osaka University Graduate School of Medicine, 2-2 (D10) Yamada-oka, Suita, Osaka 565-0871, Japan; QST Hospital, National Institutes for Quantum and Radiological Science and Technology, 4-9-1 Anagawa, Inage-ku, Chiba 263-8555, Japan; Department of Molecular Imaging and Theranostics, National Institutes for Quantum and Radiological Science and Technology, 4-9-1 Anagawa, Inage-ku, Chiba 263-8555, Japan

**Keywords:** cervical cancer, image-guided brachytherapy, hybrid brachytherapy, dose constraints, dose–volume histogram parameters

## Abstract

The aim of this study was to assess the feasibility of planning dose–volume histogram (DVH) parameters in computed tomography-based 3D image-guided brachytherapy for locally advanced cervical cancer. In a prospective multi-institutional study, 60 patients with stage IIA2–IVA cervical cancer from eight institutions were treated with external beam radiotherapy using central shielding and intracavitary or hybrid (combined intracavitary/interstitial) brachytherapy (HBT). The dose constraints were set as a cumulative linear quadratic equivalent dose (EQD2) of at least 60 Gy for high-risk clinical target volume (HR-CTV) D_90_, D_2cc_ ≤ 75 Gy for rectum, D_2cc_ ≤ 90 Gy for bladder and D_2cc_ ≤ 75 Gy for sigmoid. The median HR-CTV D_90_ was 70.0 Gy (range, 62.8–83.7 Gy) in EQD2. The median D_2cc_ of rectum, bladder and sigmoid was 57.1 Gy (range, 39.8–72.1 Gy), 68.9 Gy (range, 46.5–84.9 Gy) and 57.2 Gy (range, 39.2–71.2 Gy) in EQD2, respectively. In 76 of 233 sessions (33%), 23 patients underwent HBT, and the median number of interstitial needles was 2 (range, 1–5). HBT for a bulky HR-CTV (≥40 cm^3^) significantly improved the HR-CTV D_90_ compared with intracavitary brachytherapy alone (*P* = 0.010). All patients fulfilled the dose constrains for target and at risk organs by undergoing HBT in one-third of sessions. We conclude that the planning DVH parameters used in our protocol are clinically feasible.

## INTRODUCTION

3D image-guided brachytherapy (3D-IGBT) for locally advanced cervical cancer is gradually becoming the standard of care. This approach is replacing 2D brachytherapy and spreading throughout the world [1]. The recommendation of dose–volume histogram (DVH) parameters for 3D-IGBT planning was published by the Groupe Européen de Curiethérapie and the European Society for Radiotherapy & Oncology (GEC-ESTRO) working group [2] and the American Brachytherapy Society (ABS) [3]. The new brachytherapy technique demonstrated an improvement in local control without increasing the risk of severe complications in a multi-international prospective study [4]. Despite this, the acceptance of 3D-IGBT for cervical cancer in Japan is slower compared with Western countries [5]. One possible reason for this is that consensus-based dose constraints for high-risk clinical target volume (HR-CTV) and organs at risk (OARs) have not been well established. Since several treatment conditions in Japan are different, including the use of a central shield (CS), dose and fractionation schedule, and type of applicator [6–8], the treatment planning parameters recommended by the GEC-ESTRO working group and ABS cannot be directly applied to Japanese clinical practice. Further, GEC-ESTRO guidelines require magnetic resonance imaging (MRI) for HR-CTV and OAR delineation with applicators *in situ* at every treatment fraction [9]. Although MRI is the gold standard for 3D-IGBT planning for cervical cancer, frequent use of MRI is difficult at most institutions because of absence of MRI equipment in radiation oncology departments and because of time-consuming operational issues. Several surveys, including Japan, on the use of 3D-IGBT for cervical cancer demonstrated that computed tomography (CT) is the most commonly used imaging modality for dose specification in clinical practice [10–12].

Recently, data on the relationship between clinical outcomes and DVH parameters for 3D-IGBT based on retrospective studies on cervical cancer have been accumulated in Japan [13, 14]. In 2014, we conducted a multi-institutional prospective study (Japanese study on CT-based brachytherapy in locally advanced cervical cancer; JCBRACE) to assess the effectiveness and safety of CT-based 3D-IGBT for cervical cancer. The present study also aims to establish a consensus on the dose constraints for HR-CTV and OARs using the Japanese protocol for 3D-IGBT. Herein, we evaluated the feasibility of treatment planning DVH parameters, which were obtained based on our retrospective analyses of clinical outcomes in patients with locally advanced cervical cancer undergoing CT-based 3D-IGBT. Specifically, we attempted to assess the usefulness of newly proposed DVH parameters for brachytherapy planning.

## MATERIALS AND METHODS

### Patients

A total of 60 patients were prospectively recruited between November 2014 and November 2017 from eight institutions in Japan. Patients with histologically proven locally advanced cervical cancer, Federation of Gynecology and Obstetrics (FIGO 2008) stage IIA2–IVA, were considered suitable for curative treatment. Eligibility criteria also included patients with a tumor diameter ≥ 4 cm at initial diagnosis and no para-aortic lymph nodes ≥1 cm in minimum diameter on CT. The institutional ethical review board of each institution approved the study. Written informed consent for data acquisition was obtained from each patient. This trial is registered in the University Hospital Medical Information Network Clinical Trials Registry (UMIN-CTR; number 000016140) [15].

### Radiotherapy

The radiotherapy protocol consisted of a combination of whole-pelvis external beam radiotherapy (WP-EBRT) and high-dose-rate brachytherapy (HDR-BT). After 30 or 40 Gy of WP-EBRT, a 3–4-cm width CS was inserted [16]. For WP-EBRT, the four-field technique was utilized, and intensity-modulated radiotherapy was not allowed. Three radiotherapy schedules were provided for the protocol, which is standard in Japan [17, 18], and schedule selection was left to the attending radiation oncologist (Table 1). Boost EBRT of 6–10 Gy/3–5 fractions was performed for patients with nodal metastasis. Weekly cisplatin (40 mg/m^2^, five courses) was concurrently given with radiotherapy.

**Table 1:** Radiotherapy schedules^a^

Treatment schedule	External beam radiotherapy		HDR-BT^b^	Total EQD_2_ 10 Gy
	WP	CS		WP + HDR-BT
1	30 Gy/15 fr	20 Gy/10 fr	24 Gy/4 fr	62 Gy
2	40 Gy/20 fr	10 Gy/5 fr	18 Gy/3 fr	64 Gy
3	40 Gy/20 fr	10 Gy/5 fr	24 Gy/4 fr	72 Gy

^a^WP = whole-pelvis radiotherapy; CS = pelvis irradiation with central shielding; fr = fraction; EQD_2,_ equivalent dose in 2 Gy per fraction (the EQD_2_ is calculated using α/β = 10). ^b^Planning aim doses of HR-CTV D_90_.

HDR-BT was performed once a week using the ^192^Ir afterloading system (microSelectron-HDR; Nucletron, Elekta AB, Stockholm, Sweden). After applicator implantation, CT data were acquired with the patient in a lithotomy position or a supine position. CT slice thickness was < 3.0 mm, and CT-based treatment planning was performed. T2-weighted MR images acquired at diagnosis and just before the first brachytherapy session without an applicator were routinely used as reference images for tumor extension. There was no restriction on the type or size of applicator. Hybrid brachytherapy (HBT) combining a uterine applicator and free interstitial needles was allowed [19, 20], but interstitial brachytherapy alone was not. Ultrasound-guided insertion of interstitial needles was recommended. The contouring for HR-CTV was performed with the same definitions as the Japanese Radiation Oncology Study Group recommendations [21]. For the rectum, bladder and sigmoid, the outer organ contours were delineated. Delineation of the rectum included all regions from the anorectal junction to the rectosigmoid flexure. The dose prescription was performed according to our schedule (Table 1), whereas the source dwell patterns (i.e. times and positions) were determined based on institutional practice. In principle, plan optimization was performed according to the planning aims for HR-CTV and OARs during each brachytherapy session (Table 2).

**Table 2:** Planning aims for HR-CTV and OARs in each brachytherapy session

Treatment schedule	Dose constraint	HR-CTV D_90_	Rectum D_2cc_	Bladder D_2cc_	Sigmoid D_2cc_
1, 2	Preferable	≥7.0 Gy	<5.5 Gy	<6.5 Gy	<5.5 Gy
	limit	≥6.0 Gy	5.5–6.0 Gy	6.5–7.6 Gy	5.5–6.0 Gy
3	Preferable	≥7.0 Gy	<5.0 Gy	<6.0 Gy	<5.0 Gy
	limit	≥6.0 Gy	5.0–5.2 Gy	6.0–6.5 Gy	5.0–5.2 Gy

Total treatment doses were calculated as a cumulative linear quadratic equivalent dose (EQD2) using α/β = 10 Gy for HR-CTV and α/β = 3 Gy for OARs. The cumulative EQD2 dose was the summation of the EBRT doses (without the CS) and HDR-BT doses according to Japan Society of Gynecologic Oncology (JSGO) guidelines [18]. The final objective was a total dose in EQD2 of at least 60 Gy corresponding to HR-CTV D_90_, D_2cc_ ≤ 75 Gy for rectum, D_2cc_ ≤ 90 Gy for bladder and D_2cc_ ≤ 75 Gy for sigmoid. Table 2 shows the DVH parameters as planning aims for HR-CTV and OARs in each brachytherapy session. Dose optimization was performed manually and targeted to fulfil the ‘preferable’ or ‘limit’ values. The first priority for dose optimization was to fulfill a total dose in EQD2 of D_2cc_ ≤ 75 Gy for rectum, D_2cc_ ≤ 90 Gy for bladder and D_2cc_ ≤ 75 Gy for sigmoid based on retrospective studies using the Japanese standard schedule and the EMBRACE study [4–5, 22–26]. The threshold value of each group was determined by an expert discussion based on the guidelines and the results of the retrospective study in Japan.

### Quality assurance

To improve the quality of this study, workshops and annual monitoring were established. The workshop was conducted to homogenize treatments between institutions. A radiation oncologist, a medical physicist and a radiation technologist from each institution participated in the workshop. A dummy run was performed to ensure that the selected medical professionals were suitable for 3D-IGBT treatment planning. All case report forms were checked at the data center. If there was any doubt, the data center queried the relevant institution and consulted with the study office. Moreover, to ensure treatment plan integrity, the data center checked the relationship between the total reference air kerma and isodose surface volume.

### Analysis

DVH parameters for target and OARs were analyzed as a total treatment dose and each 3D-IGBT dose. The treatment plan quality of each brachytherapy session was evaluated and classified according to the criteria shown in Table 2. When a dose for OARs did not meet the ‘limit’ level, it was classified as ‘unsatisfied’. All treatment plans were divided into two volume groups: the non-bulky group (HR-CTV < 40 cm^3^) and the bulky group (HR-CTV ≥ 40 cm^3^). Moreover, the relationship between the HR-CTV volume groups and DVH parameters (HR-CTV D_90_, bladder D_2cc_, rectum D_2cc_ and sigmoid D_2cc_) was evaluated in intracavitary brachytherapy (ICBT) and HBT. Statistical analyses were performed using JMP® 14 software (SAS Institute Inc., Cary, NC, USA) and *P* values were calculated using the Wilcoxon rank-sum test. The level of statistical significance was defined as *P* < 0.05. For HBT, the relationship between the number of needles and the HR-CTV and the percentage of needle dwell time to total dwell time were evaluated.

## RESULTS

### Patient demographics

Patient demographics and tumor characteristics are shown in Table 3. The HR-CTV width as defined on MRI had a median size at initial presentation of 5.2 cm (range, 3.1–7.3 cm) and a median size just before the first brachytherapy session of 3.6 cm (range, 0–6.3 cm). Two patients had a complete response on MRI just before the first brachytherapy session.

**Table 3:** Patient demographics^a^

Variable	Median (range)	*n* (%)
Age (years)	53 (26–73)	
BMI (kg/m^2^)	21.0 (16.3–35.1)	
Performance score (ECOG)		
0		43 (72)
1		16 (27)
2		1 (2)
Histology		
Squamous cell carcinoma		56 (93)
Adenocarcinoma		3 (5)
Adenosquamous carcinoma		1 (2)
FIGO Stage (2008)		
IIA2		4 (7)
IIB > 4 cm		39 (65)
IIIA		1 (2)
IIIB		16 (27)
Pelvic lymph node		
Negative		31 (52)
Positive		29 (48)
Tumor size at initial presentation (cm)	5.2 (3.1–7.3)	
Width < 5 cm		24 (40)
Width ≥ 5 cm		36 (60)
Tumor size just before first brachytherapy (cm)	3.6 (0–6.3)	
Width < 5 cm		50 (83)
Width ≥ 5 cm		10 (17)
Tumor volume at first brachytherapy (cm^3^)	32.4 (12.3–117.4)	
<40 cm^3^		36 (60)
≥40 cm^3^		24 (40)

^a^BMI = body mass index; ECOG = Eastern Cooperative Oncology Group.

### Treatment characteristics

Table 4 shows the treatment characteristics. A total of 50 patients (83%) were treated with schedule 1 (WP 30 Gy + CS 20 Gy + HDR-BT 4 fractions). All patients received at least one cycle of cisplatin with EBRT, and 27 patients had a radiation boost to involved lymph nodes. A total of 233 brachytherapy sessions were performed, in which 76 treatment sessions (33%) were HBT. One or two needles were used for 53 treatment sessions (70%).

**Table 4:** Treatment characteristics

Variable	*n* (%)
Radiotherapy schedule^a^	
Schedule 1 (WP 30 Gy + CS 20 Gy + HDR-BT 4 fr)	50 (83)
Schedule 2 (WP 40 Gy + CS 10 Gy + HDR-BT 3 fr)	7 (12)
Schedule 3 (WP 40 Gy + CS 10 Gy + HDR-BT 4 fr)	3 (5)
Central shield width	
3 cm	57 (95)
4 cm	3 (5)
EBRT nodal boost	
Yes	27 (45)
No	33 (55)
Brachytherapy applicators	
Tandem + ovoid	214 (92)
Tandem	11 (5)
Ovoid	2 (1)
Cylinder	6 (3)
Brachytherapy technique	
Intracavitary (ICBT)	157 (67)
Intracavitary/interstitial (HBT)	76 (33)
Number of needles used in HBT	
1	26 (34)
2	27 (36)
3	14 (18)
4	7 (9)
5	2 (3)
Number of HBT for each session	
First	19 (32)
Second	22 (37)
Third	20 (33)
Fourth	15 (28)

^a^WP = whole-pelvis external beam radiotherapy; fr =fraction.

### DVH parameters of the treatment plan

Table 5 shows the total treatment doses for each DVH parameter. The median HR-CTV D_90_ was 70.0 Gy (range, 62.8–83.7 Gy) in EQD2 (α/β = 10). The median D_2cc_ of rectum, bladder and sigmoid was 57.1 Gy (range, 39.8–72.1 Gy), 68.9 Gy (range, 46.5–84.9 Gy) and 57.2 Gy (range, 39.2–71.2 Gy) in EQD2 (α/β = 3), respectively. All patients fulfilled the final objective of a total dose in EQD2 of at least 60 Gy for HR-CTV D_90_, D_2cc_ ≤ 75 Gy for rectum, D_2cc_ ≤ 90 Gy for bladder and D_2cc_ ≤ 75 Gy for sigmoid.

**Table 5:** DVH parameters for HR-CTV and OARs. Doses are shown as WP + HDR-BT in EQD2 dose. The EQD_2_ is calculated using α/β = 10 for HR-CTV and α/β = 3 for OAR

Variable^a^	Median (range)
HR-CTV (EQD_2_ 10 Gy)	
D_98_	62.4 Gy (51.6–76.2)
D_90_	70.0 Gy (62.8–83.7)
D_50_	102.6 Gy (89.4–119.3)
Rectum (EQD_2_ 3 Gy)	
D_0.1cc_	74.7 Gy (42.9–105.7)
D_2cc_	57.1 Gy (39.8–72.1)
Bladder (EQD_2_ 3 Gy)	
D_0.1cc_	89.9 Gy (57.4–119.3)
D_2cc_	68.9 Gy (46.5–84.9)
Sigmoid (EQD_2_ 3 Gy)	
D_0.1cc_	71.9 Gy (45.2–89.7)
D_2cc_	57.2 Gy (39.2–71.2)
Point A (EQD_2_ 10 Gy)	67.2 Gy (60.9–95.0)
Rectum D_ICRU_ (EQD_2_ 3 Gy)	61.3 Gy (40.2–121.1)
Bladder D_ICRU_ (EQD_2_ 3 Gy)	50.7 Gy (35.5–89.4)

^a^WP = whole-pelvis external beam radiotherapy; EQD_2_ = equivalent dose in 2 Gy per fraction; ICRU = International Commission on Radiation Units and Measurements.

**Fig. 1.**
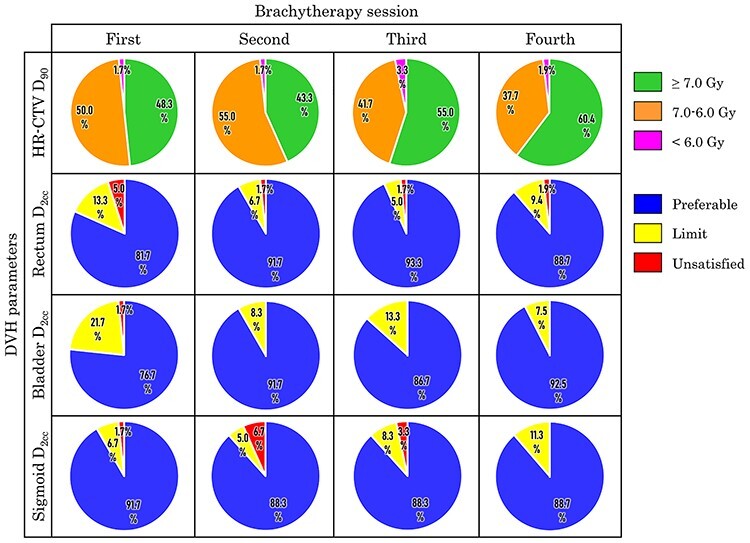
Evaluation of treatment plan quality among each brachytherapy session, which was classified according to the criteria shown in Table 2. When the ‘limit’ dose level was not met in organs at risk, it was classified as ‘unsatisfied’.

The treatment plan quality in each brachytherapy session is shown in Figure 1. The median HR-CTV in each brachytherapy session was 32.4, 30.2, 28.3 and 28.8 cm^3^ for the first, second, third and fourth session, respectively. As the brachytherapy session progressed, the proportion of HR-CTV D_90_ ≥ 7.0 Gy increased. The proportion of ‘preferable’ and ‘limit’ levels accounted for 219 of 233 sessions (94%). The proportion of ‘unsatisfied’ in each brachytherapy session was 2.6% (6 of 233) for rectum, 0.4% (1 of 233) for bladder and 3.0% (7 of 233) for sigmoid. Two or more ‘unsatisfied’ did not occur in the same session. In sigmoid, the proportion of ‘unsatisfied’ of 6% [4 of 67] in the HR-CTV ≥ 40 cm^3^ group was higher than that in the HR-CTV < 40 cm^3^ group 1.8% [3 of 166] and higher than that in the other OARs (rectum 3.0% [2 of 67] and bladder 0% [0 of 67]).

**Fig. 2.**
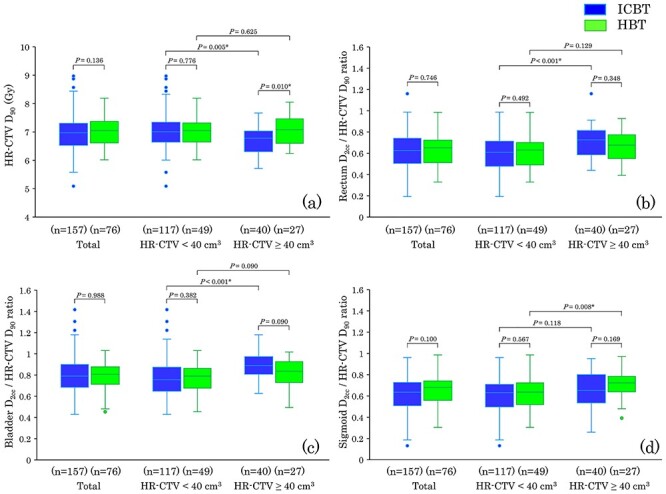
A box plot shows the DVH parameter relationships among ICBT, HBT and HR-CTV. The 50% interquartile range, median and range of data are displayed. All treatment plans were divided into two volume groups: the non-bulky group (HR-CTV < 40 cm^3^) and the bulky group (HR-CTV ≥ 40 cm^3^). (**a**) HR-CTV D_90_ and HR-CTV volume. (**b**) The dose ratio of rectum D_2cc_/HR-CTV D_90_ and HR-CTV volume. (**c**) The dose ratio of bladder D_2cc_/HR-CTV D_90_ and HR-CTV volume. (**d**) The dose ratio of sigmoid D_2cc_/HR-CTV D_90_ and HR-CTV volume.

Figure 2 shows the relationships between HR-CTV and DVH parameters for ICBT and HBT. For ICBT, the HR-CTV D_90_ in the HR-CTV ≥ 40 cm^3^ group was significantly lower compared with the HR-CTV D_90_ in the HR-CTV < 40 cm^3^ group (*P* = 0.005; Fig. 2a). In addition, the dose ratios of rectum D_2cc_/HR-CTV D_90_ and bladder D_2cc_/HR-CTV D_90_ were significantly higher in the HR-CTV ≥ 40 cm^3^ group compared with the HR-CTV < 40 cm^3^ group (*P* < 0.001; Fig 2b and c). For HBT, the dose ratio of sigmoid D_2cc_/HR-CTV D_90_ was significantly higher in the HR-CTV ≥ 40 cm^3^ group compared with the HR-CTV < 40 cm^3^ group (*P* = 0.008; Fig. 2d). There was no significant difference in the HR-CTV D_90_ between the HR-CTV ≥ 40 cm^3^ group and the HR-CTV < 40 cm^3^ group (*P* = 0.625). The dose ratios of rectum D_2cc_/HR-CTV D_90_ and bladder D_2cc_/HR-CTV D_90_ were not significantly different between the groups. The HR-CTV D_90_ of HBT in the HR-CTV ≥ 40 cm^3^ group was significantly higher compared with that of ICBT (*P* = 0.010).

### Contribution of interstitial needles

**Fig. 3.**
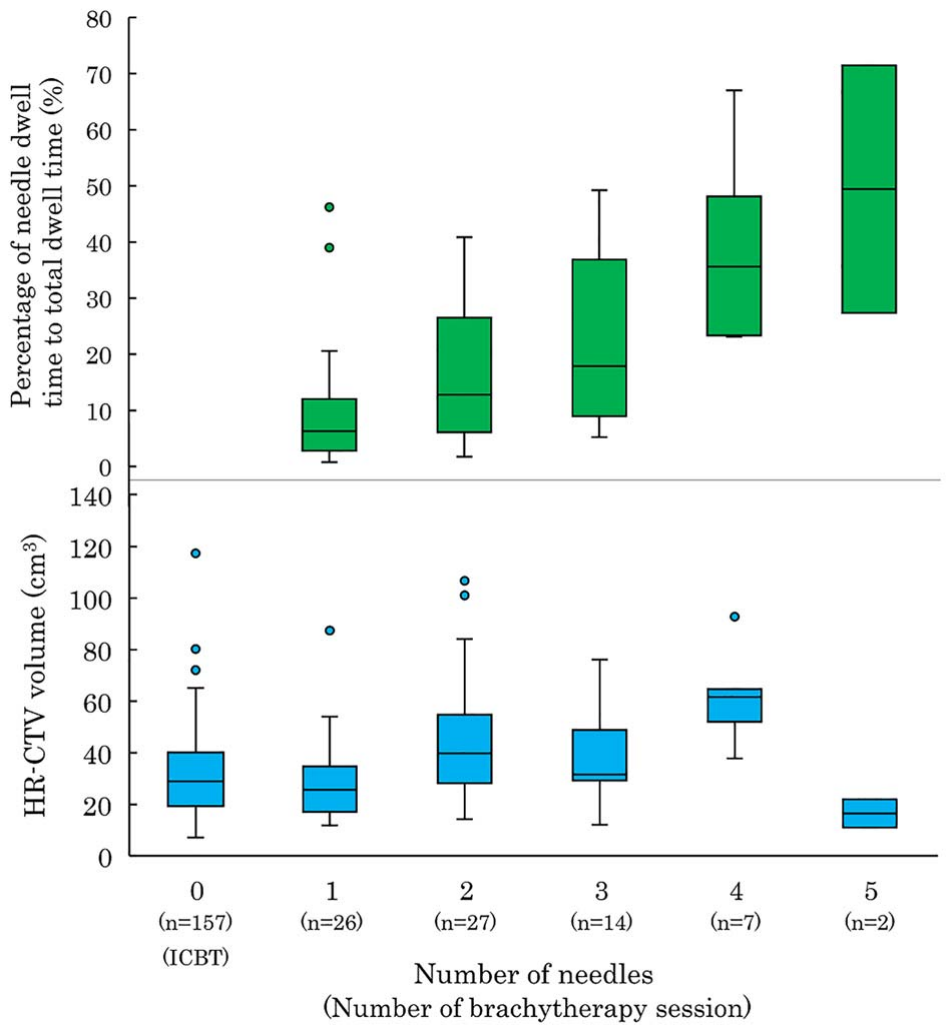
A box plot showing the relationship between HR-CTV, needle contribution and number of needles. The 50% interquartile range, median and range of data are displayed.

In 76 HBT sessions undergone by 23 patients, the median number of inserted needles was 2 (range, 1–5). The relationships among HR-CTV, number of needles, and percentage of needle dwell time to total dwell time are shown in Fig. 3. The median percentage dose delivered from the needles was 12.7% (range, 0.8–71.5%). The contribution of the percentage of needle dwell time to total dwell time tended to increase with the number of needles. By contrast, a clear relationship was not confirmed between the number of needles and HR-CTV.

## DISCUSSION

The present study was designed to assess the feasibility of treatment planning DVH parameters and to establish a consensus on the dose constraints for target and OARs in CT-based 3D-IGBT for cervical cancer among Japanese institutions. All patients achieved the final objective total dose in EQD2 of at least 60 Gy for HR-CTV D_90_, D_2cc_ ≤ 75 Gy for rectum, D_2cc_ ≤ 90 Gy for bladder and D_2cc_ ≤ 75 Gy for sigmoid by undergoing HBT for 32.6% of 3D-IGBT sessions. ‘Preferable’ and ‘limit’ dose levels accounted for 219 of all 233 sessions (94%). Therefore, our results confirm that DVH parameters based on the retrospective analyses for target and OARs in CT-based 3D-IGBT for locally advanced cervical cancer are feasible in a prospective setting and that the proposed planning aims in each 3D-IGBT session appear to be useful in clinical practice. Importantly, clinical effectiveness and safety will be confirmed by the emerging clinical results of this study in the near future.

In the dose prescription of 3D-IGBT, the physician must decide on a specific balance between HR-CTV and OARs doses. Although dose constraints of total dose in EQD2 for the rectum, bladder and sigmoid are important to avoid severe late complications, the significance of planning aims for HR-CTV and OARs in each 3D-IGBT session has not previously been sufficiently clarified. Okazaki *et al*. suggested that 90 or 98% of the HR-CTV should be covered with at least 6.5 Gy (~9 Gy in EQD2) or 5.5 Gy (~7 Gy in EQD2) per session to obtain favorable local control using a Japanese treatment regimen [14]. The dose–response relationship between dosimetric parameters and local control should be analyzed in our study. The planning aims used in the current study (Table 2) were flexible because physicians had three to four opportunities for dose optimization over the course of 3D-IGBT. For example, even when the rectal dose resulted in an overdose beyond the ‘limit’ dose level in the first session, the physician could reduce the rectal dose below the ‘preferable’ dose level from the second session onward with tumor shrinkage. With this approach, total dose constraints were kept within D_2cc_ ≤ 75 Gy for the rectum. The clinical significance and flexibility in our planning aims should be further evaluated.

The threshold dose of HR-CTV D_90_ in our study was determined based on the clinical outcome of Japanese studies. Previous retrospective studies from Japanese institutions have shown that the median HR-CTV D_90_ ranged from 65 to 74 Gy in EQD2 [14, 23]. Murakami *et al*. reported that both local control and progression-free survival were significantly favorable in patients receiving ≥60 Gy for HR-CTV D_90_ in EQD2 [23]. In our study, the median HR-CTV D_90_ was 70.0 Gy (range, 62.8–83.7 Gy) in EQD2, which is comparable to previous Japanese studies, but lower than other studies [27–30]. One of the major reasons for this was that the dose contribution from the CS was completely ignored according to JSGO guidelines [18]. Tamaki *et al*. demonstrated that 24–56% of CS doses contributed to the HR-CTV D_90_ when the CS had a width of 3 cm, and 13–35% of CS doses contributed to HR-CTV D_90_ when the CS had a width of 4 cm in a phantom study [31]. Also, Tamaki *et al*. suggested that the addition of 5–7 Gy to the D_90_ value may account for the dose contribution from the CS. Since the actual tumor volume, shape and location may vary during treatment, precise estimation of the dose contribution of the CS is difficult. Another possible reason for the lower HR-CTV D_90_ is the difference in the imaging modality (MRI vs CT) used for treatment planning. Viswanathan *et al*. reported that CT-based tumor contours can overestimate the tumor width significantly compared with MRI, resulting in significant differences in the HR-CTV D_90_ as well as volume treated with respect to the prescription dose or greater for the HR-CTV [32]. In our study, however, T2-weighted MR images acquired at diagnosis and just before the first brachytherapy session without an applicator were routinely used as reference images for tumor extension. Comprehensive discussions of the reason for the relatively lower total dose of HR-CTV D_90_ in the Japanese treatment regimen will be required to accompany the emerging clinical outcomes.

Compared with ICBT, HBT is advantageous in that it can optimize the anatomy-oriented dose, which results in an improvement in DVH parameters. As shown in Fig. 2, HBT maintained target coverage for bulky tumors (HR-CTV ≥ 40 cm^3^) without increasing the relative dose of the bladder D_2cc_ and rectum D_2cc_ to HR-CTV D_90_. Anderson *et al*. reported that DVH parameter improvements in the HR-CTV volume ≥ 40 cm^3^ group were diminished even with 3D-IGBT when a conventional intracavitary applicator is used without supplementary needles [33]. Chargari *et al*. also reported that a lower ability to reach target HR-CTV D_90_ planning and an HR-CTV volume ≥ 40 cm^3^ correlated with a high propensity of relapse, with these factors being interrelated [34]. Therefore, HBT has an essential role in achieving the final objective total dose, especially in the HR-CTV volume ≥ 40 cm^3^ group.

One of the limitations of our study is that the decision criteria for HBT were not provided in the protocol and the selection of ICBT or HBT was left to the attending radiation oncologist. Several authors have summarized the needle position and number of needles for applicators with needle holes, such as Utrecht applicators [35, 36]. Due to the limited availability of applicators dedicated to HBT, free needles are commonly used in Asian countries [19, 37–39]. Liu *et al*. claimed that HBT using free needles is clinically feasible for large-volume tumors (>5 cm) [38]. Yoshida *et al*. performed a simulation analysis and proposed that HBT should be considered for a HR-CTV > 4 × 3 × 3 cm (36 cm^3^) [39]. In our study, HBT was performed even in the group with a small HR-CTV, and there was no clear regularity in the number of needles (Fig. 3). It is assumed that there was inter-physician variability. Further study of HBT standardization is needed in terms of the volume and shape of HR-CTV and the anatomical relationship between HR-CTV and OARs.

In conclusion, in this multi-institutional prospective study on CT-based 3D-IGBT for locally advanced cervical cancer, all 60 patients fulfilled the dose constrains for HR-CTV D_90_ and D_2cc_ for the rectum, bladder and sigmoid, indicating that the treatment planning DVH parameters were feasible. Additionally, our planning aims for HR-CTV and OARs, which were achievable in > 90% of 3D-IGBT sessions, would be useful in brachytherapy planning. Indications and the dosimetric impact of HBT should be further evaluated to effectively balance HR-CTV and OAR doses.
